# Effect of Diosmin Administration in Patients with Chronic Venous Disorders on Selected Factors Affecting Angiogenesis

**DOI:** 10.3390/molecules24183316

**Published:** 2019-09-12

**Authors:** Marcin Feldo, Magdalena Wójciak-Kosior, Ireneusz Sowa, Janusz Kocki, Jacek Bogucki, Tomasz Zubilewicz, Jan Kęsik, Anna Bogucka-Kocka

**Affiliations:** 1 Department of Vascular Surgery and Angiology, Medical University of Lublin, Staszica 11, 20-081 Lublin, Poland; tomasz.zubilewicz@umlub.pl (T.Z.); jankesik@umlub.pl (J.K.); 2 Department of Analytical Chemistry, Medical University of Lublin, Chodźki 4a, 20-093 Lublin, Poland; kosiorma@wp.pl (M.W.-K.); i.sowa@umlub.pl (I.S.); 3 Department of Clinical Genetics, Medical University of Lublin, Lublin, Poland, Radziwiłłowska 11, 20-080 Lublin, Poland; janusz.kocki@umlub.pl (J.K.); jacekbogucki@wp.pl (J.B.); 4 Chair and Department of Biology and Genetics, Medical University of Lublin, W. Chodźki 4A, 20-093 Lublin, Poland; anna.bogucka-kocka@umlub.pl

**Keywords:** diosmin, chronic venous disorders, angiostatin, vascular endothelial growth factor, tumor necrosis factor alpha, interleukin 6, fibroblast growth factor 2, plasminogen

## Abstract

Diosmin is a natural compound with a wide range of biological activity, e.g., it improves lymphatic drainage, supports microcirculation, and increases venous tone, and venous elasticity, hence, it is applied in the pharmacotherapy of chronic venous disorders (CVD). The aim of this study was to assess the correlation between diosmin administration (2 × 600 mg daily) in patients suffering from CVD and the levels of selected factors influencing angiogenesis, which are involved in CVD pathophysiology. Thirty-five CVD patients were examined. Levels of plasma tumor necrosis factor alpha (TNF alpha), vascular endothelial growth factor (VEGF-A and VEGF-C); angiostatin, interleukin 6 (IL-6), fibroblast growth factor 2 (FGF2); and plasminogen (PLG) were measured with an Elisa assay before and after three months of diosmin administration. The clinical symptoms of CVD were monitored using ultrasound images, echo Doppler assay, visual analogue scale (VAS), and measurement of the leg circumference. The average content of TNF alpha, VEGF-C, VEGF-A IL-6, and FGF2 decreased after the therapy with diosmin in a significant manner; with *p* < 0.001, *p* < 0.05, *p* < 0.05, *p* < 0.01, and *p* < 0.01, respectively, and a significant (*p* < 0.05) increase in the plasma angiostatin level after the three-month treatment was found. A significant (*p* < 0.05) decrease in edema and the average leg circumference of the patients was observed after the therapy. Diosmin influences the angiogenic and inflammatory mechanisms involved in the pathophysiology of edema presented in patients with a different class of CVD.

## 1. Introduction

Diosmin is a natural compound from the flavonoid class commonly found in citrus fruit and in the Rutaceae family. It possesses a wide range of biological activity; however, its effect on the cardiovascular system has the greatest significance from the point of view of human health. It has been shown that diosmin improves lymphatic drainage, supports microcirculation, increases venous tone, venous elasticity, and capillary resistance, and reduces capillary filtration and capillary hyperpermeability [1,2,3,4,5]. It also exerts an anti-inflammatory effect and alleviates oxidative stress [6], hence, it is considered to be an important therapeutic tool in the strategy of chronic venous disorders (CVD) treatment.

CVD is a disorder related to functional and morphological abnormalities of the venous system. Edema, skin lesions, and structural changes in the vein wall, such as varicose veins and venous leg ulcers, are the most common symptoms of CVD with prevalence between 20 and 60% [7,8]. Incompetent venous valves cause blood reflux, which results in increased and sustained venous hypertension and is considered the primary stage of CVD. Venous hypertension increases the hydrostatic pressure in superficial subcutaneous veins and capillaries, which leads to transcapillary filtration with excessive lymphatic flow causing edema formation [9,10]. This has a considerable impact on quality of life. According to clinical–etiology–anatomy–pathophysiology (CEAP), classification of CVD includes several clinical stages C0–C6. C0 shows no visible signs, C1 is manifested as teleangiectasies or spider veins, C2 is presented by varicose veins, C3 is associated with edema. C4 shows skin changes (pigmentation or lipodermatosclerosis), C5 stage shows healed skin ulcers and C6 active skin ulcers [8].

Skin lesions in advanced stages of CVD associated with increased proliferation of skin capillaries and microcirculatory abnormalities may be a result of a disturbed level of factors responsible for angiogenic response, such as vascular endothelial growth factor (VEGF), fibroblast growth factor 2 (FGF2) and angiostatin. VEGF is a family of proteins comprising five members; however, two of them, i.e., VEGF-A and VEGF-C, have a major impact on CVD pathology. They stimulate the formation of vessels and increase the permeability of endothelial cells by the formation of vesiculo–vacuolar organelles, providing the transport of plasma proteins such as fibrinogen and plasminogen from the blood stream into the surrounding tissue [11]. On the other hand, FGF2 is a pleiotropic heparin-bonded factor that promotes proliferation of a broad spectrum of cell types. FGF2 activity is characterized by pro-angiogenic phenotype in endothelial cells leading to the neovascularization process and exerts a potent angiogenic response in a variety of animal models [12]. 

In turn, angiostatin is a potent inhibitor of angiogenesis. It can be generated from circulating plasminogen by various proteases that are active in blood neutrophils and perivascular space (such as MMP-3, -7, -12). Angiostatin is a specific factor affecting only endothelial cells. It inhibits the proliferation and migration of endothelial cells and the formation of the endothelial cell tube as a result of inhibition of plasmin generation. Angiostatin also stimulates the apoptosis of endothelial cells. Moreover, it blocks microvascular formation and decreases the permeability of vessels [13,14]. Plasminogen (PLG), as a precursor for angiostatin formation, may be also involved in CVD pathology. Additionally, in CVD patients, an increase in the hydrostatic pressure and blood stasis in capillaries prompt leukocyte adhesion to the capillary endothelium and trigger inflammatory cascades involving various types of inflammatory mediators [15].

There are many studies on the beneficial effects of diosmin administration in CVD and it has been proved that diosmin improves the quality of life of patients with CVD [8,16]; however, the biological activity of this flavonoid is still a subject of interest for many researchers, and intensive investigations are still being conducted to find the molecular mechanism of diosmin action. The aim of our study was to investigate the correlation between diosmin administration in patients suffering from CVD and the levels of selected factors influencing angiogenesis, because some reports suggest a role of the pro-angiogenic factor VEGF in CVD pathology [16,17,18]. The level of pro-inflammatory cytokines including TNFα and IL-6 was monitored as well, as they may induce blood vessel formation by direct engagement of endothelial cells or indirectly by inducing them to produce angiogenic mediators [19].

## 2. Results

### 2.1. Clinical Symptoms

The estimation of clinical symptoms allowed division of the patients with CVD into the following classes: C2—varicose vein; C3—varicose vein and edema; C4—varicose vein, edema, and skin lesions. Initially, ultrasound examination (echo-Doppler assay) was performed to assess the changes in the blood flow in the veins during the compression maneuver [20]. The recorded spectral signal of the flow curve revealed a reverse flow, which was longer than one second, during the compression maneuver in each patient with CVD included in the study (Figure S1). This sign called “reflux” is associated with incompetent venous valves. 

Based on the ultrasound images, the patients with thrombosis (Figure S2b) were excluded from the clinical trial because venous insufficiency in these patients may have had a genetic background, and our study was focused on CVD with unknown etiology. 

Perivascular spaces in the subjects were also monitored to observe pathological symptoms associated with edema. In 13 patients, no extravasation of edema fluid through the endothelium into the perivascular space was observed in the ultrasound images; therefore, these patients were classified into the C2 group. Fluid in the perivascular space was visible in 22 subjects. In this group, six patients with skin lesions were classified as C4 and the others were assigned to the C3 group.

**Figure 1:**
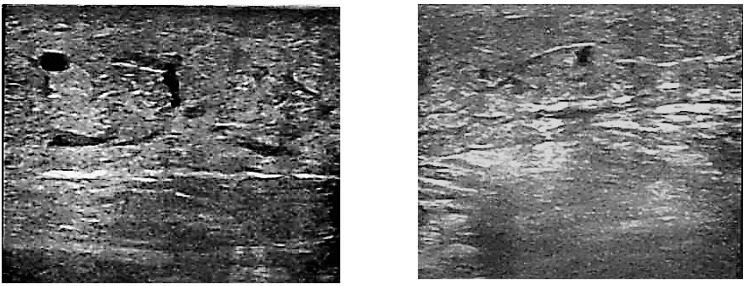
Ultrasound images of the perivascular space in a patient before (**a**) and after (**b**) 3 months of diosmin treatment.

After the three-month treatment with diosmin, no changes were noted in the echo-Doppler assay; however, a reduction in the extravasation of edema fluid into the perivascular space was observed in 78% of patients from C3 and 83% of patients from C4 groups. This resulted in a decrease in edema, and the average leg circumference in the patients decreased from 30.74 (±0.58) cm to 29.11 (±0.39) cm in a significant manner (*p* < 0.05). Examples of perivascular space images before and after the diosmin treatment are shown in Figure 1a,b, respectively.

Pain, assessed on the VAS scale, was absent in 10 (28.5%) participants of our study. The other 25 subjects declared feeling minor pain ranging from 1 to 3 on the VAS scale and, after three months of diosmin administration, less severe pain was declared by 19 patients in this group. Lack of pain reduction was observed in six patients. The differences between the C2, C3, and C4 groups were statistically insignificant.

To summarize, the relief of CVD symptoms allowed us to transfer 13 patients from the C3 to the C2 class.

### 2.2. Basic Blood Parameters 

Factors affecting the venous circulation and indicators of inflammation were investigated to exclude patients with an excessive level of triglycerides, acute phase plasma protein, and creatinine. Moreover, these parameters were also monitored after 3 months of the treatment with diosmin to assess the possible changes. The results are summarized in Table 1.

The values obtained for each patient were within the acceptable range and no significant changes (*p* < 0.05) in the level of the investigated factors were observed in patients after treatment with diosmin.

### 2.3. Monitoring the Level of TNF Alpha, IL-6, VEGF-A, VEGF-C, FGF2, Plasminogen and Angiostatin in Plasma

**Figure 2:**
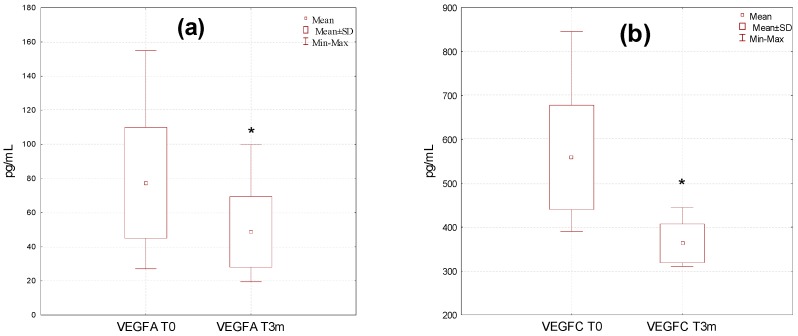

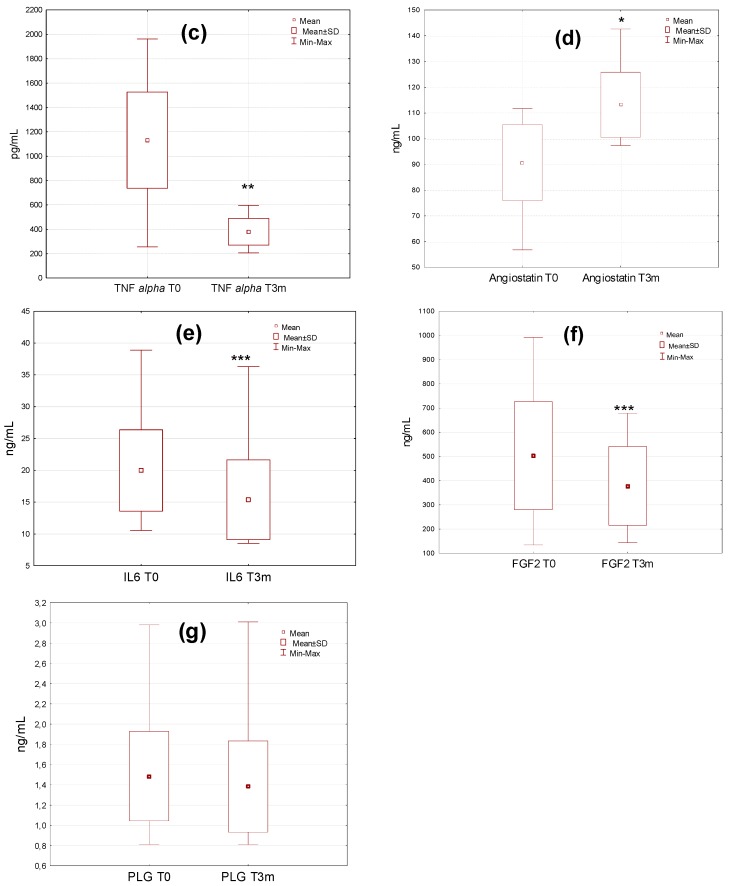
Plasma level of VEGF-A (**a**), VEGF-C (**b**), TNF alpha (**c**), angiostatin (**d**), IL6 (**e**), FGF2 (**f**) and plasminogen (**g**) before (T0) and after three months (T3m) of diosmin treatment. * *p* < 0.05, ** *p* < 0.001, *** *p* < 0.01.

A high variation in the levels of the investigated factors affecting angiogenesis was observed in the studied group, particularly before the diosmin treatment (Figure 2). Values were in the range of 27.1–154.8 pg/mL, 395.1–862.3 pg/mL, 258.1–1988.8 pg/mL, 57.2–115.2 ng/mL, 11.2–38.9 ng/mL, 133.3–990.1 ng/mL, and 0.81–2.99 ng/mL for VEGF-A, VEGF-C, TNF alpha, angiostatin, IL6, FGF2, and plasminogen, respectively. The dispersion of the results was reduced after the therapy, which was particularly evident for VEGF-C (322.8–444.3 pg/mL) and TNF alpha (201.2–599.8 pg/mL).

The average content of VEGF-A, VEGF-C, TNF alpha, IL-6, and FGF2 decreased in a significant manner after the therapy with diosmin, with *p* < 0.05, *p* < 0.05, *p* < 0.001, *p* < 0.01, and *p* < 0.01, respectively, whereas a significant (*p* < 0.05) increase in plasma angiostatin was found. No significant differences in the serum concentration of plasminogen after three months of treatment with diosmin were observed.

**Figure 3:**
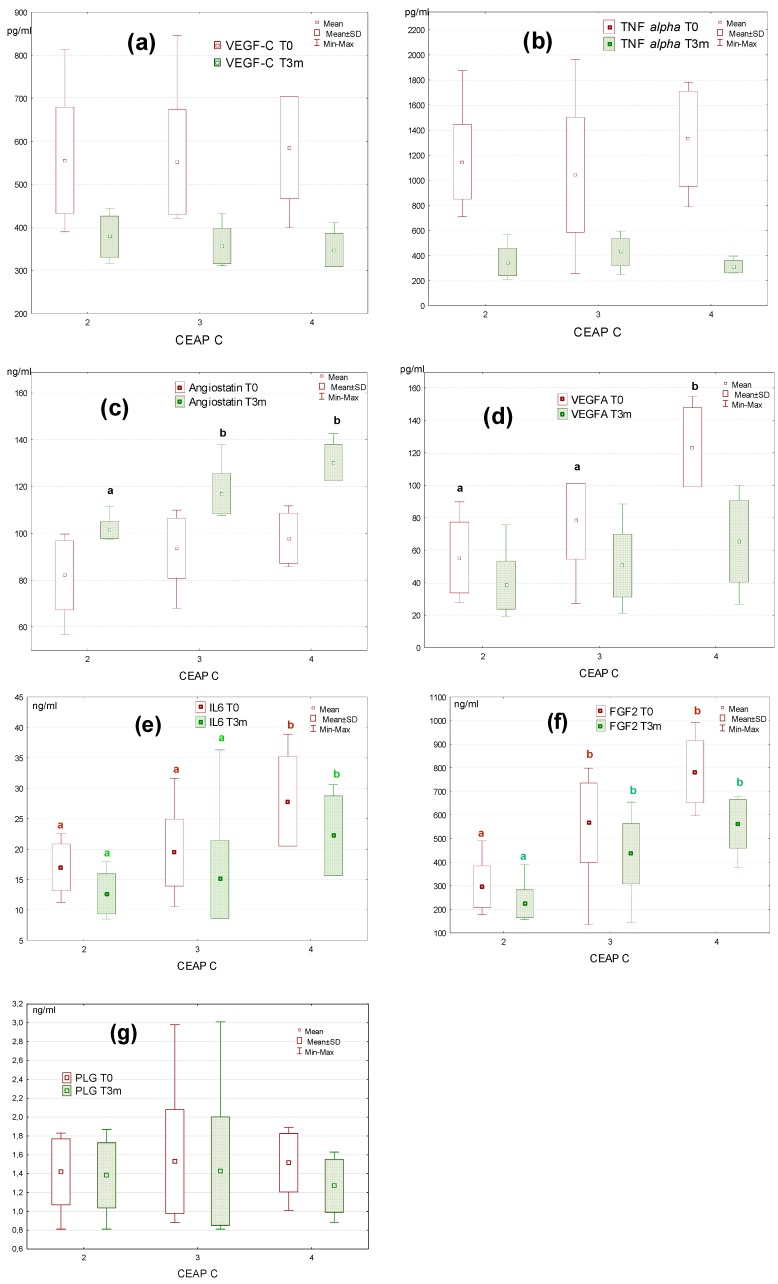
Comparison of the levels of the investigated factors in the C2, C3, and C4 groups before (T0) and after three months of diosmin treatment (T3m). The data followed by the different letters are statistically significant: (**a**) and (**b**) lack of statistically significant differences (*p* > 0.05), (**c**) *p* = 0.005 for C2 and C3; *p* = 0.0001 for C2 and C4; (**d**) p = 0.05; (**e**) *p* = 0.01 for C2 and C3; *p* = 0.001 for C2 and C4; (**f**) *p* = 0.01 for C2 and C3; *p* = 0.001 for C2 and C4.

The analysis of the data from individual classes of CVD patients revealed no statistically significant differences in the level of VEGF-C, TNF alpha, angiostatin, and plasminogen between C2, C3, and C4 patients before the diosmin treatment (Figure 3a–c,g). In the case of VEGF-A, significant differences were only demonstrated between C4 and C2 (*p* = 0.05), as well as C4 and C3 (*p* = 0.05) before the therapy (Figure 3d). The plasma level of IL6 and FGF2 differed in a significant manner between the investigated classes of patients both before and after diosmin administration. For IL6, statistically significant differences were observed between C2 and C4 (*p* = 0.001) and between C3 and C4 (*p* = 0.01) (Figure 3e). Meanwhile, for FGF2 differences were noted between C2 and C3 (*p* = 0.01), as well as between C2 and C4 (*p* = 0.0001) class of patients (Figure 3f). After diosmin administration, differences between groups in the level of VEGF-C, TNF alpha, and plasminogen were statistically insignificant. For angiostatin, the differences between C2 and C3 (*p* = 0.0005) as well as C2 and C4 (*p* = 0.0001) were noted (Figure 3c). In each class of patients, a decline in VEGF-C, TNF alpha, and VEGF-A, as well as an increase in angiostatin levels were found.

Changes in the level of investigated parameters in patients before and after therapy with diosmin were assessed by correlation analyses performed using Spearman’s test (*p* < 0.05, s < 0.05) (Figure S3). It revealed significant correlations between VEGF A T0/T3m and angiostatin T0/T3m.

## 3. Discussion

The beneficial effects of diosmin treatment on improvement of the quality of life in CVD, such as a decrease in the leg circumference and alleviation of pain, have been described in numerous reports, and the results obtained in this study confirm this observation. However, the investigation of factors involved in CVD pathology such as IL-6, TNF alpha, FGF2 and VEGF can help to explain the mechanism of diosmin activity. Our study proved that the three-month oral administration of 600 mg of diosmin twice daily significantly diminished the plasma level of the selected pro-inflammatory and pro-angiogenic factors, e.g., TNF alpha, IL-6, FGF2, VEGF-A, and VEGF-C. It could affect the diminished interstitial edema, the reduced inflammatory stage and pain, and the downregulation of VEGF-related processes.

Increased expression of total VEGF has been found in patients with CVD compared to the control group, which confirms its role in the development of abnormalities of the venous system [18]. VEGF-C can stimulate lymphangiogenesis and regulates the growth of lymphatic vessels. Vasodilatation and increased vascular permeability cause transcapillary filtration that exceeds lymphatic flow and forms interstitial edema via mechanisms involving VEGF. Moreover, TNF alpha and VEGF are involved in the regulation of multiple steps of angiogenesis [21] and the synergistic action of FGF and VEGF in angiogenesis has been reported as an essential regulatory mechanism in this process [12]. Moreover, angiogenesis induced by FGF is often blocked by VEGF inhibition suggesting that FGF controls angiogenesis upstream of VEGF by modulating VEGF function [22]. FGF and VEGF may contribute to the capillary proliferation in advanced CVD but it is not clear whether it is hypoxia induced or directly related to capillary proliferation. It is known that in advanced forms of CVD (C4, C5) capillary convolution and proliferation are prominent features [23].

Angiogenesis is an unfavorable process in venous system disorders that can result in skin lesions in advanced stages of CVD. In turn, activated leukocytes can release a large amount of elastase and other proteinases associated with tissue injury and lipodermatosclerotic skin remodeling [24]. Proteinase release by leukocytes/neutrophils may contribute to the generation of angiogenesis inhibitors such as angiostatin. Dysregulation of angiogenesis caused by local imbalance between pro-angiogenic and anti-angiogenic molecules is associated with pathological conditions of CVD [21,24]. On the other hand, FGF2 level is elevated at the sites of chronic inflammation, tissue injury and in human cancer because inflammatory cells including mononuclear cells, T lymphocytes and mast cells increase the expression of FGF2 it has been also proved that FGF2 may amplify endothelial cells response to inflammatory stimuli [12]. Howlader et al. observed higher plasma levels of VEGF in patients with higher range of edema symptom and then concluded that VEGF levels correlated with CEAP stage and sensation of swelling [25].

The pain mechanism in the venous disease is focused on local inflammation related to pro-inflammatory mediators released locally by leukocytes and activated receptors in the venous wall [26,27]. In previous research, the positive correlation between venous stasis (decreased blood flow) and IL-6 was observed [28] and it suggests the potential role of IL-6 as an important factor of the inflammatory cascade related to variety of vascular diseases [29]. Unmyelinated C fibers are present in varicose vein walls remodeled by chronic inflammation, and these nervous fibers play a key role in the onset of pain [27,30]. The pain is not constantly present in CVI symptomatology. It can be absent in 45% of subjects with reflux on Doppler lower limb venography and is independent of the varicose vein volume [27]. In our study, minor pain was declared by 71.5% of patients. After three months of the treatment with diosmin, reduction of pain accompanied by reduced edema was noted. 

On the other hand, a significant increase in the angiostatin plasma level was observed in the patients after three months of diosmin administration. This increase was accompanied by the reduction of VEGF levels. In our study, no significant changes in plasminogen serum level was observed. The increase of angiostatin level may suggests enhanced cleavage of plasminogen as a source of this peptide. In this context we could conclude, that 3-months diosmin administration could influence on the plasminogen synthesis in order to replenish cleaved part. On the other hand, increased angiostatin serum level could interfere into VEGF-PAI-uPA interplay and finally plasminogen level did not change.

Summarizing, the three-month diosmin administration modulates pro-inflammatory as well as pro- and anti-angiogenic mechanisms in CVD pathology. As a pharmacological vasoactive agent, diosmin can protect CVD patients against edema progression by inhibition of inflammatory pathways, simultaneously influencing pro-angiogenic/anti-angiogenic balance by an increase of anti-angiogenic factor and reducing the level of pro angiogenic factors. 

One of the limitations of this study is that the patients did not use compression therapy stockings due to individual and autonomic patient decisions. A potential limitation is that the number of patients was relatively small, but on the other hand, the study group was homogeneous.

## 4. Materials and Methods 

### 4.1. Selection of Patients and Administration of Diosmin 

The study was approved by the Independent Ethics Committee of the Medical University of Lublin. Written informed consent was obtained from all study participants. Between April 2014 and June 2016, 38 patients who had a history of primary CVD and met the selection criteria for the pharmacological treatment of CVD were screened for the study at the Department of Vascular Surgery and Angiology, Medical University of Lublin. The exclusion criteria included diabetes, autoimmune disease, tumor, renal insufficiency, liver disease, recent surgery or trauma, prior vein surgery, deep/superficial vein thrombosis, congestive heart failure, and pregnancy. The inclusion criterion was the presence of CVD of the C2, C3 or C4 degrees in the CEAP classification with bilateral incompetent GSV (greater saphenous vein) in the femoral and calf region classified as Ep (primary). None of the patients were treated with diuretics or vasoactive drugs for at least three months prior to the recruitment date. They declared not to have used diuretics or compression therapy during the study period. The recruited patients had received compression therapy before but found it “intolerable” or “irritant”. From the initial group of 38 patients, three patients did not complete the therapy (reasons unknown) and they were excluded from the study. Detailed patient characteristics are shown in Table 2. 

The patients received 2 × 600 mg/day of diosmin (Phlebodia, Laboratoires Innothera, Arcueil, France). They were instructed not to change their daily habits such as diet and daily rhythm of work and rest. Checkup visits were scheduled every 30 days. All assays were carried out before (T0) and after three months (T3m) of the therapy. The patients declared no side effects after the diosmin administration. 

### 4.2. Echo-Doppler Examination 

Echo-Doppler examination of the leg venous system was performed in all the patients using a Toshiba Aplio 500 echoscanner (Canon Medical System Europe) with color-flow Doppler and a 5 MHz linear transducer [31]. The images of Doppler venography were analyzed separately by two radiologists. The patients were classified according to the detailed CEAP—Anatomic classification—As (s—superficial, 2 and 3—great saphenous vein) and Pathophysiologic classification—Pr (r-reflux). Compression maneuver was performed with the patient in standing position in order to maintaining adequate venous distension. Evaluation of great saphenous vein reflux by duplex imaging was performed using a 7–10 MHz linear probe located on calf and then on medial thigh level. Compression of the vein distally to the probe location shows a spike venous flow velocity as the blood is pushed anterograde, toward the heart.

### 4.3. Oedema Assessment 

Edema was assessed by measuring the circumference of both legs (in cm) using a tape [32] fixed in the ankle region, 8 cm above the central point of the lateral malleolus and 13 cm from the floor in order to increase the reproducibility of the measurement. The procedure was done separately by two independent observers in the same temperature conditions and at the same time of day—at 10:00 a.m.

### 4.4. Evaluation of Pain 

Pain was assessed using a 10 cm visual analogue scale (VAS) from 0 (no pain) to 10 cm (intolerable pain).

### 4.5. Blood Collection 

Blood samples were collected in tubes (Sarstedt Monovette EDTA KE, Nümbrecht, Germany) containing EDTA (1 mg/mL) and immediately centrifuged at 1500 g for 10 min at 4 °C (Eppendorf centrifuge 5702). Supernatant plasma was separated and stored at –20 °C until the measurement. 

### 4.6. Biochemistry Assay 

Levels of TNF alpha, VEGF-C (Thermo Fischer, Waltham, MA, USA), VEGF-A (PromoCell GmbH, Germany), angiostatin IL-6, plasminogen and FGF2 (Elabscience, Houston, TX, USA) were measured using an enzyme-linked immunoabsorbent assay kit (ELISA). The procedures were performed according to instruction of the manufacturer.

### 4.7. Statistical Analysis

Statistical analysis of the results (Statistica 10 software, StatSoft Polska) was carried out using descriptive statistics and the following parameters: Group size (N), arithmetic mean, median, minimum and maximum values of variables, and standard deviation (SD). Comparison between the study groups was made using a Kruskal-Wallis multiple comparison test (non-parametric one-way analogue of ANOVA). Correlation analyses were performed using Spearman’s test. 

## Figures and Tables

**Table 1:** Average values (±SD) of selected physiological parameters in patients before (T0) and after 3 months of diosmin treatment (T3m).

Parameter	T0	T3m
Total cholesterol [mg/dL]	198.3 (±8.3)	199.8 (±8.3)
Triglycerides [mg/dL]	113.77 (±32.3)	108.7 (±29.9)
CRP [mg/dL]	3.7 (±1.1)	3.6 (±1.03)
Complement C3 [mmol/L]	1.46 (±0.3)	1.45 (±0.38)
Complement C4 [mmol/L]	0.43 (±0.18)	0.41 (±0.19)
Fibrinogen [mg/dL]	318.4 (±82.9)	331 (±90.8)
D-dimer [μg/L]	414.9 (±128.8)	378.5 (±100.5)
BMI	26.1 (±1.6)	26.08 (±1.7)
Creatinine [mg/dL]	0.92 (±0.27)	0.89 (±0.22)
Albumin [g/dL]	4.50 (±1.56)	4.47 (±0.97)
Sodium [mmol/L]	139.20 (±1.24)	140.20 (±1.38)

There were no significant differences between variables.

**Table 2:** Demographic data of recruited patients (n = 35).

Age (mean ± SD)	49 ± 10.8
Sex (male/female)	17/18
Smokers	4
CEAP Class	C2: 13
	C3: 16
	C4: 6
Comorbidities	Hypertension: 5
	Coronary disease: 1
Medical treatment	Β-blockers: 2
	Anti-platelet: 1
	Statins: 1

## Supplementary Materials

The following are available online, Figure S1: Echo-Doppler assay obtained in patients without (a) and with (b) blood reflux, Figure S2: Ultrasound images of patients’ veins without (a) and with thrombosis marked in red (b) Figure S3: Correlation between investigated parameters (Spearman’s test).
