# Supplementary material for: Effect of Diosmin Administration in Patients with Chronic Venous Disorders on Selected Factors Affecting Angiogenesis
Source: Molecules. 2019 Sep 12;24(18):3316. doi: 10.3390/molecules24183316 (PMC6767141; doi:10.3390/molecules24183316)
Supplement: Supplementary file 1 [file molecules-24-03316-s001.pdf]

Supplementary

Effect of diosmin administration in patients with chronic venous disorders on selected factors affecting angiogenesis

Marcin Feldo[1],*, Magdalena Wójciak-Kosior[2], Ireneusz Sowa[2], Janusz Kocki[3], Jacek Bogucki[3], Tomasz Zubilewicz[1], Jan Kęsik[1], Anna Bogucka-Kocka[4]

[1] Department of Vascular Surgery and Angiology, Medical University of Lublin, Staszica 11, 20-081 Lublin, Poland; martinf@interia.pl (M.F.), tomasz.zubilewicz@umlub.pl (T.Z.), jankesik@umlub.pl (J.K.)

[2] Department of Analytical Chemistry, Medical University of Lublin, Chodźki 4a, 20-093 Lublin, Poland; kosiorma@wp.pl (M.W.K); i.sowa@umlub.pl (I.S)

[3] Department of Clinical Genetics, Medical University of Lublin, Lublin, Poland, Radziwiłłowska 11, 20-080 Lublin; janusz.kocki@umlub.pl (J.K.); jacekbogucki@wp.pl (J.B.)

[4] Chair and Department of Biology and Genetics, Medical University of Lublin, W. Chodźki 4A, 20-093 Lublin, Poland, anna.bogucka-kocka@umlub.pl

* Correspondence: martinf@interia.pl; Tel.: +48 81532 5707

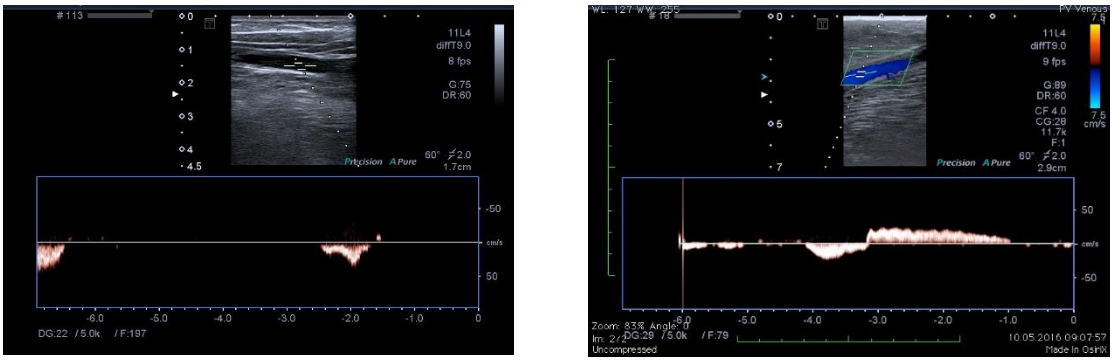

(a) (b)

Figure S1. Echo-Doppler assay obtained in patients without (a) and with (b) blood reflux.

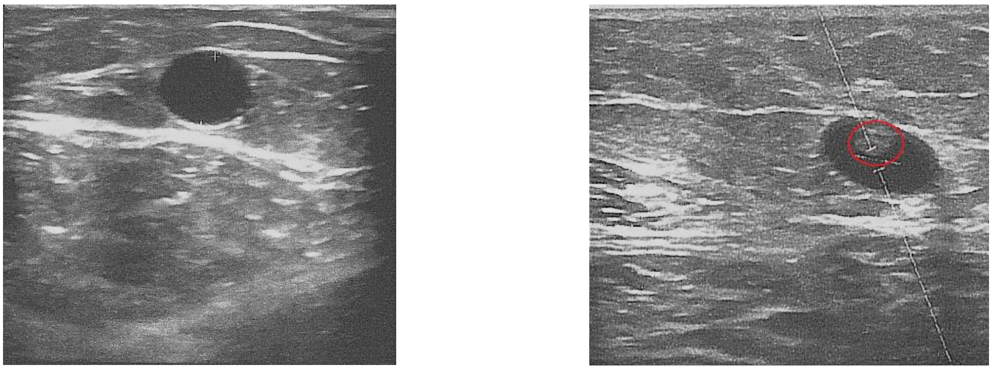

(a) (b)

Figure S2. Ultrasound images of patients' veins without (a) and with thrombosis marked in red (b).
